# Assessment of Public Perception and Attitude Toward Skin Donation in Saudi Arabia

**DOI:** 10.7759/cureus.29289

**Published:** 2022-09-18

**Authors:** Fawwaz F AlShammrie, Lubna H Aloufi, Sarah S Aldharman, Manahel S Almutairi, Mohammed H Al Mansour, Lara S Alyahiwi, Maram M Alhati, Reema A Aldawish

**Affiliations:** 1 Dermatology, University of Hail, Hail, SAU; 2 College of Medicine, Princess Norah Bint Abdulrahman University, Riyadh, SAU; 3 College of Medicine, King Saud Bin Abdulaziz University for Health Sciences, Riyadh, SAU; 4 College of Medicine, University of Hail, Hail, SAU; 5 Otolaryngology - Head and Neck Surgery, Najran General Hospital, Najran, SAU; 6 College of Medicine, Sulaiman AlRajhi University, Al Bukairiyah, SAU

**Keywords:** general population, saudi srabia, attitude, perception, skin donation

## Abstract

Background

The skin is the largest organ of the body. Burns are important health issues that significantly affect a variety of population groups. Skin grafting is still regarded as the gold standard in surgical burn treatment. The availability of skin for grafting is one of the main challenges in burn surgical therapies. Thus, this study aimed to assess the public perception and attitude regarding skin donation in Saudi Arabia.

Method

The study was a cross-sectional study that included the general population in Saudi Arabia (≥ 18 years old). A self-administered survey was distributed online. Statistical analysis was carried out using RStudio (R version 4.1.1). Categorical data were presented as frequencies and percentages, while continuous data were expressed as the median and interquartile range (IQR).

Results

A total of 8515 were included in the study. Most participants (71.5%) were females. Among the respondents, 64.9% were between the ages of 18 and 30. Females had a higher knowledge level of skin graft donation compared to males. Participants aged >30 years had lower knowledge levels compared to younger participants. Lower knowledge scores were also observed among married, widows, as well as uneducated participants. Five thousand two hundred and seven (61.1%) participants support skin donation. The most reported barrier to skin donation was religious reasons (52.4%), while the main motives for supporting skin donation were humane-related factors (73.2%). The influence of close relatives on participants' decisions to donate was evident in 52.6% of the instances.

Conclusion

It was found that most participants support skin donation in Saudi Arabia. Religious reasons were the most common hindering factors against skin donation. Females and young-aged groups were found to have a higher knowledge level. Further studies are recommended to shed light on this subject allowing for appropriate solutions implantation.

## Introduction

Burn is described as damage to the skin or other tissues and categorized into several types depending on the etiology, such as flame or scald, chemical, and electrical burns. Burns are a major public health issue that is estimated to be responsible for 180,000 deaths annually [1,2]. Only a single donor’s tissues may provide benefits for up to 100 patients [3]. While solid organs of a cadaver may save the lives of less than 10 patients, tissue procurement can save much more lives and improve the quality of life in many other patients. Unfortunately, neither the medical community and government health systems nor the media have addressed tissue donation as organ donation and transplantation [4]. Other organs, such as the liver and kidney, can withstand more than 60% damage, but losing 40% of one's skin can be fatal unless the tissue is replaced [5]. It has been reported that, after road accidents, burns are the second-largest category of injuries [6]. One of the most often employed strategies in burn surgery is skin grafting [7]. No blood group, age, or color matching is needed because everyone's skin is conceivable [8].

Compared to high-income countries, the rate of child burn mortality is currently approximately seven times greater in low- and middle-income countries [2]. Some burns are severe enough to require medical attention [9]. Most burns were not fatal, but they had a significant impact on the patient's quality of life and the financial stability of their family due to extended hospital stays and functional loss, which resulted in social isolation and disgrace [10,11]. Depending on the degree and proportion of burns, it is determined whether hospitalization is necessary or whether outpatient care is appropriate. Deep burns will often necessitate surgical debridement and skin grafting. The therapeutic application of allograft skin in burn wound area was first defined in 1938 when Bettman replicated his success in the treatment of children with significant full-thickness burns [12].

By preserving a sufficient supply of allograft skin, the use of skin banks in industrialized countries has considerably reduced the incidence of burn mortality in both children and adults [12,13]. Therefore, skin grafts can be used to protect against severe burns with extensive total body surface area to reduce the risk of infection-related mortality. The graft is obtained from a donor (any unburned spot of the same patient and this is referred to as an autograft, or a cadaveric skin which is known as allograft, or from animals, which is known as xenograft). The success of a skin graft take is determined by a variety of parameters, including nutrition transport and the extent of vascularity from the recipient bed [14,15].

Several published studies investigate the general public's views and beliefs about organ donation. In the United States, a study was conducted in 1985 to see how individuals with open minds might think about organ donation [16]. The results showed that people would undoubtedly donate their kidneys (50%) as opposed to merely donating their skin (40%) [16]. Similar findings were reported in an Iranian study among high school students, which found that kidneys (88%) and hearts (84%) were the most often donated organs while skin (51%) was the least donated [17]. However, a study conducted among medical professionals in Nigeria revealed that 25.5% of them were willing to donate their skin after death [18]. A previous local study in Riyadh, Saudi Arabia was conducted to investigate the public knowledge, awareness, and attitude toward skin donation [19]. It included 698 participants and found that most participants were willing to donate their skin and were aware of skin donation aspects [19]. To further evaluate their findings, we sought to obtain a larger sample size that is more representative of the Saudi population. Given the significance of skin donation and the paucity of relevant literature in Saudi Arabia, the purpose of this study was to assess the public perception and attitude regarding skin donation in Saudi Arabia.

## Materials and methods

A descriptive cross-sectional study was conducted in Saudi Arabia between March 2022 and June 2022. The target subjects were the general population in Saudi Arabia. A self-administered questionnaire was used to collect the data. An informed consent form was provided to all participants before filling out the questionnaire. The questionnaire was provided electronically using Google Forms. The data were entered into Microsoft Excel (Microsoft Corporation, New Mexico, USA) and subsequently uploaded and analyzed using RStudio (R version 4.1.1). Continuous variables were reported as mean ± SD, while categorical variables were reported using frequencies and percentages. All information was confidential and was only used for scientific research, and participation in this research was voluntary and optional with informed consent on the first page. The data analysis and publication process did not require any identifiable personal data. This research study protocol was submitted to the research ethical committee at the University of Hail and was approved with the approval number H-2022-284.

Inclusion criteria and exclusion criteria

The general population in Saudi Arabia (≥18 years old) was included in this study. Participants who did not fill out the whole questionnaire and aged less than 18 years were excluded from the study.

Sampling technique and sample size calculation

OpenEpi® version 3.01 software was used to estimate our sample size which is representative of Saudi Arabia's population of 34 million. The representative sample size required is 385, with a margin error determined as 5% and a confidence level determined as 95%. We aimed to obtain more than the calculated sample size to overcome potential exclusions. Non-probability convenience sampling techniques have been used.

Data collection instrument and procedures

The questionnaire was designed by the authors after an extensive review of the relevant literature. Then it was reviewed by experts in the field of dermatology to ensure clarity and simplicity that all items in the questionnaire were relevant for the study purpose. The experts were contacted via emails and required to rate the relevance of each item in each section using a three-point Likert scale (1 = not relevant, 2 = somewhat relevant, 3 = relevant) and to suggest other items that might not have been considered. The survey was designed in Arabic to make it easier for the population to read and understand. The results of the survey were translated to English using accredited translation tools. The final questionnaire was then pretested for content on 25 participants of different demographic characteristics, using its electronic version to ensure its coherence and wording. The participants who participated in the pretest were not included in the analysis. The developing questionnaire contained three sections that involved questions related to demographic information, perception, and attitude toward skin donation. The first page of the questionnaire was designated for informed consent. An electronic Google Form survey was used and distributed on different social media platforms such as WhatsApp, Twitter, and Telegram. To obtain a large number of participants in our study, a team of data collectors from different regions in Saudi Arabia has been recruited to collect the data from their respective regions. Each data collector was provided with a copy of the questionnaire to collect the responses and then submitted it through the electronic form for data analysis.

Calculation of the knowledge score

The correct answers to 11 items in the public awareness domain were considered in the calculation of knowledge scores. These items had a total of 28 correct answers. Therefore, an overall raw score was computed by summing up the correct responses, and the score ranged between 0 and 28 for each participant. To facilitate the interpretation of scores, a percentage score was calculated by using the following formula: percentage score = (raw score/28) * 100. 

Statistical analysis

Statistical analysis was carried out using RStudio (R version 4.1.1). Categorical data were presented as frequencies and percentages, while continuous data were expressed as the median and interquartile range (IQR). Factors associated with the percentage knowledge score were assessed by conducting a univariate linear regression analysis. The significantly associated factors were further entered into a multivariate regression model to assess the independent association with knowledge. Results of the regression analysis were expressed as beta coefficients and their respective 95% confidence intervals (95% CIs). A p-value of 0.05 indicated statistical significance.

## Results

Demographic information of the participants 

Initially, we received the responses of 8,630 participants. However, 8,515 respondents agreed to participate in the current study with a response rate of 98.7%. More than half of the respondents aged 18-30 years (64.9%), were single (59.8%), and had obtained a bachelor's degree (59.6%). More than two-thirds of the participants were females (71.5%). The demographic characteristics of the participants are shown in Table 1.

**Table 1:** Demographic information of the participants

Parameter	Category	N (%)
Age (years)	18-30	5,528 (64.9%)
	31-40	1,475 (17.3%)
	41-50	1,001 (11.8%)
	More than 50	511 (6.0%)
Gender	Male	2,423 (28.5%)
	Female	6,092 (71.5%)
Marital status	Single	5,088 (59.8%)
	Married	2,999 (35.2%)
	Widow	148 (1.7%)
	Divorced	280 (3.3%)
Education	Public education	2,600 (30.5%)
	Bachelor	5,073 (59.6%)
	Postgraduate studies	704 (8.3%)
	Uneducated	138 (1.6%)

Public perception toward skin donation

The most commonly perceived sources from which a skin graft could be obtained included the same person (57.7%) and another person who has the same skin color (21.6%). The majority of respondents (70.5%) indicated that the skin of hidden areas (abdomen, thigh, etc.) can be a candidate source for skin grafting. Significant proportions of the participants had no awareness regarding skin storage after donation (55.4%), the duration for which skin grafts could be preserved (65.8%), the number of types of skin grafts (63.9%), the obstacles that could prevent the application of skin donation in KSA (31.2%), and the possibility of using animal skin for grafting (44.5%). More details about participants’ responses to the perceptions items are listed in Table 2.

**Table 2:** Participants’ public perception toward skin donation *Indicates a correct response.

Parameter	Category	N (%)
What is the source from which the skin can be taken when donating?	From the same person*	4,910 (57.7%)
	From another person who has similar skin color*	1,843 (21.6%)
	From another person who has different skin color*	262 (3.1%)
	From animals*	289 (3.4%)
	I do not know	1,211 (14.2%)
What is the area from which the skin is taken in the donor body?	Abdomen skin*	519 (6.1%)
	The skin of the peripheries*	556 (6.5%)
	The skin of the face*	186 (2.2%)
	The skin of hidden areas such as the abdomen and thigh*	6,004 (70.5%)
	I do not know	1,250 (14.7%)
Can the skin be stored after donation?	No	1,357 (15.9%)
	Yes*	2,444 (28.7%)
	I do not know	4,714 (55.4%)
For how long is it possible to preserve the skin graft?	Cannot keep skin	994 (11.7%)
	Two days	728 (8.5%)
	7-8 days*	694 (8.2%)
	8-14 days	495 (5.8%)
	I do not know	5,604 (65.8%)
How many types of skin grafts are there?	One	542 (6.4%)
	Two	1,219 (14.3%)
	Three*	1,317 (15.5%)
	I do not know	5,437 (63.9%)
From your point of view, what are the most important obstacles that prevent the application of skin donation in the Kingdom of Saudi Arabia?	Low numbers of available donors	2,364 (27.8%)
	Lack of experience in the region	1,353 (15.9%)
	High costs	916 (10.8%)
	Religious rulings for donation	1,225 (14.4%)
	I do not know	2,657 (31.2%)
How do you think the outcomes of skin grafting can be?	Completely different from the skin near to it	756 (8.9%)
	Unrecognizable because it is identical to the adjacent skin	1,065 (12.5%)
	Similar but can be distinguished from the skin near to it*	4,535 (53.3%)
	I do not know	2,159 (25.4%)
With time, does the difference between skin graft and the skin near to it diminish and it becomes difficult to differentiate between them?	No	1,462 (17.2%)
	Yes*	3,848 (45.2%)
	I do not know	3,205 (37.6%)
Is it possible to use animal skin for skin graft?	No	2,077 (24.4%)
	Yes*	2,651 (31.1%)
	I do not know	3,787 (44.5%)

**Figure 1:**
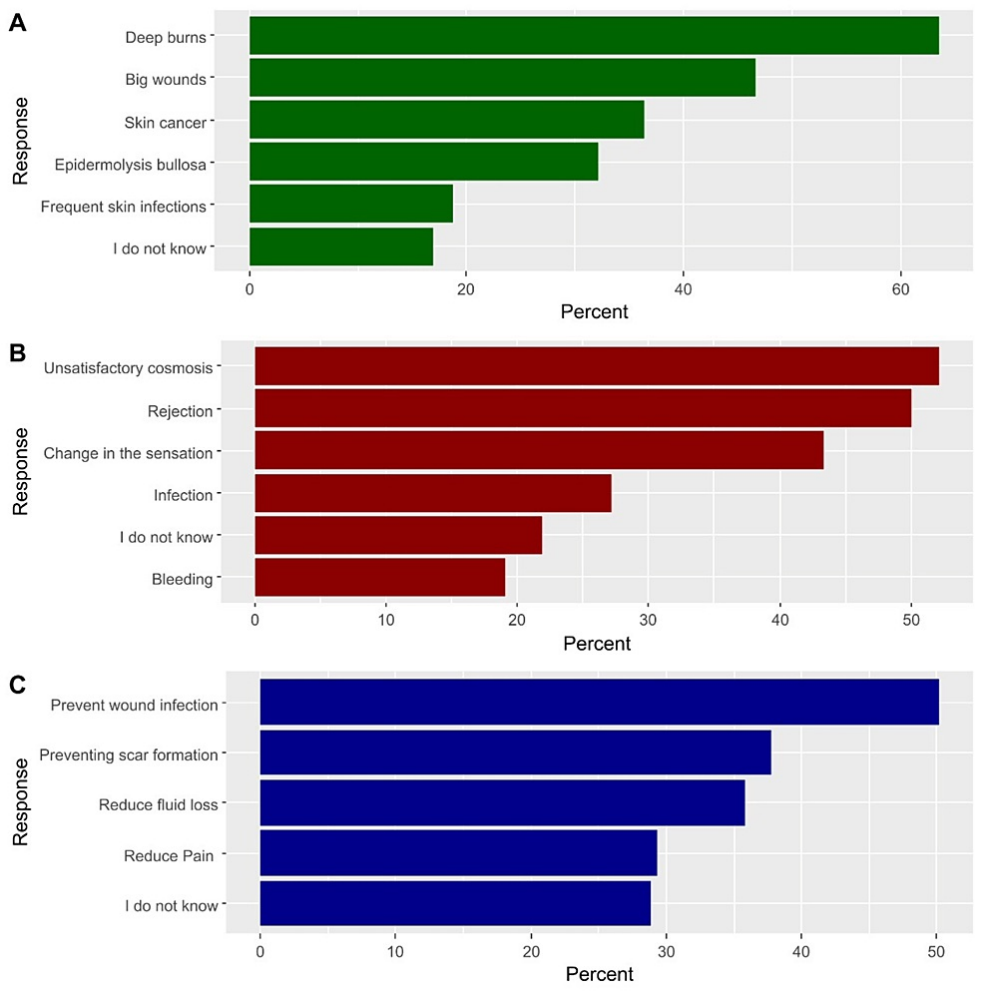
The percentage of participants’ responses to questions of multiple responses, including the lesions which may require skin grafting (A), the potential complications that may develop after grafting (B), and the benefits of grafting (C).

Regarding the items with available multiple responses, results demonstrated that the most commonly perceived conditions that require skin grafting were deep burns (63.5%) and big wounds (46.6%). Additionally, the most frequently reported complications of post-grafting were unsatisfactory esthetic outcomes (52.1%) and rejection of allogeneic skin graft by the recipient (50.1%). On the other hand, the most commonly reported benefits of skin grafting were the prevention of wound infection (50.1%) and the prevention of scar formation (37.8%). Figure 1 illustrates the percentage of participants’ responses to questions of multiple responses.

The knowledge score and the factors associated with participants’ knowledge 

The median (IQR) percentage score of participants’ awareness was 32.14 (21.43-42.86). Based on the univariate regression analysis, the female gender was significantly associated with higher knowledge scores compared to males (β = 2.19, 95%CI, 1.47-2.90, p < 0.001). Nevertheless, participants aged more than 30 years had consistently lower scores than their younger counterparts, including the participants aged 31-40 years (β = -3.24, 95%CI, -4.10 to -2.37, p < 0.001), 41-50 years (β = -4.28, 95%CI, -5.29 to -3.26, p < 0.001), and >50 years (β = -4.88, 95%CI, -6.25 to -3.51, p < 0.001). Lower knowledge scores were also apparent among married participants (β = -3.53, 95%CI, -4.22 to -2.85, p < 0.001), widows (β = -5.77, 95%CI, -8.24 to -3.30, p < 0.001), and divorced participants (β = -3.39, 95%CI, -5.21 to -1.57, p < 0.001), as well as uneducated respondents (β = -7.76, 95%CI, -10.4 to -5.16, p < 0.001). 

The above-reported significant variables were subsequently entered into a multivariate regression model. Results showed independent associations between lower knowledge scores and participants aged >30 years (β = -1.84, 95%CI, -2.91 to -0.76, p < 0.001 for those aged 31-40 years, β = -2.66, 95%CI, -3.93 to -1.39, p < 0.001 for participants aged 41-50 years, and β = -2.73, 95%CI, -4.32 to -1.14, p < 0.001 for participants aged >50 years), married (β = -1.85, 95%CI, -2.82 to -0.89, p < 0.001) and widowed participants (β = -3.46, 95%CI, -6.07 to -0.85, p = 0.009), as well as uneducated respondents (β = -6.08, 95%CI, -8.69 to -3.46, p < 0.001). Conversely, females had independently higher scores than males (β = 1.92, 95%CI, 1.20-2.63, p < 0.001). Table 3 illustrates factors associated with participants’ knowledge about skin grafting. 

**Table 3:** Factors associated with participants’ knowledge about skin grafting 95%CI, 95% confidence interval.

Parameter	Category	Univariate			Multivariate		
		Beta	95%CI	p-Value	Beta	95%CI	p-Value
Age	18-30	Ref	Ref		Ref	Ref	
	31-40	-3.24	-4.10, -2.37	<0.001	-1.84	-2.91, -0.76	<0.001
	41-50	-4.28	-5.29, -3.26	<0.001	-2.66	-3.93, -1.39	<0.001
	More than 50	-4.88	-6.25, -3.51	<0.001	-2.73	-4.32, -1.14	<0.001
Gender	Male	—	—		—	—	
	Female	2.19	1.47, 2.90	<0.001	1.92	1.20, 2.63	<0.001
Marital status	Single	Ref	Ref		Ref	Ref	
	Married	-3.53	-4.22, -2.85	<0.001	-1.85	-2.82, -0.89	<0.001
	Widow	-5.77	-8.24, -3.30	<0.001	-3.46	-6.07, -0.85	0.009
	Divorced	-3.39	-5.21, -1.57	<0.001	-1.45	-3.40, 0.50	0.146
Education	Public education	Ref	Ref		Ref	Ref	
	Bachelor	-0.02	-0.74, 0.69	0.946	0.21	-0.51, 0.93	0.566
	Postgraduate studies	-0.98	-2.24, 0.29	0.129	0.80	-0.49, 2.09	0.227
	Uneducated	-7.76	-10.4, -5.16	<0.001	-6.08	-8.69, -3.46	<0.001

Attitudes toward skin donation

In general, out of the 8,515 respondents, 5,207 participants (61.1%) declared that they support skin donation after death, whereas 3,307 participants (38.8%) did not support skin donation (one missing value). Among the non-supporters, religious reasons were the most commonly reported hindering factors, followed by other reasons (15.0%) and social reasons (21.1%). Contrastingly, humane-related factors were the primary motives for supporting skin donation (73.2%) followed by religious motives (24.9%). The impact of close individuals on participants’ decisions to donate was apparent among 52.6% of the respondents. In the instance of losing a part of the skin, the majority of respondents stressed that they would go to the skin bank. Furthermore, 72.0% of the respondents agreed to replace a lost part of the skin in a visible place with another part in a hidden area. Table 4 illustrates participants’ attitudes toward skin donation.

**Table 4:** Participants’ attitudes toward skin donation *Results are based on 3307 participants who did not support skin donation after death; otherwise, results are based on 5207 participants who supported donation. ¥The responses are based on a multiple-response item.

Parameter	Category	N (%)
In the case of your refusal to donate the skin after death, what is the reason for your refusal?*	Religious reasons	1,732 (52.4%)
	Social reasons	697 (21.1%)
	Cosmetic reasons	380 (11.5%)
	Other	498 (15.0%)
What is your primary motive for donating skin?^¥^	Religious	1,294 (24.9%)
	Humane	3,809 (73.2%)
	Financial	121 (2.3%)
If someone close to you needs to donate the skin, does it affect your decision?	Yes	2,737 (52.6%)
In case you lose a part of your skin for any reason:	I will go to the skin bank	4,321 (83.0%)
	I do not mind my skin look remaining the same	886 (17.0%)
If you lose a part of the skin in a visible place, would you mind transferring a piece of hidden skin area to replace the skin of the face or hands in case they are injured?	Yes	3,747 (72.0%)

## Discussion

Limited literature has investigated the public perception of skin donation in Saudi Arabia. The present study aimed to assess the perception and attitude regarding skin donation among the general population and to identify the associated factors. This is the first large-scale study investigating the public perception and attitude regarding skin donation in Saudi Arabia. More than half of the participants, 5,207 (61.1%), were in the support of skin donation after death. A similar finding was found in another study in which the majority of the respondents, 546 (78.22%), supported the idea of organ donation to a skin bank after their death [19]. A similar result was also shown in an Indian study in which the willingness to become a future donor was demonstrated by 205 (57%) participants [20].

The current study findings showed that a large proportion of the participants, 5,604 (65.8%), were not aware of the skin graft preservation time frame. Only 694 (8.2%) participants knew that skin can be preserved for 7-8 days, and it was found in the literature that the maximum preservation times indicated were 7-8 days [21].

Regarding the number of types of skin grafts, more than half of the participants, 5,437 (63.9%), were not aware of them. However, 1,317 (15.5%) were aware that there are at least three types, which is consistent with the findings of a review conducted that skin grafts are classified primarily into full-thickness skin grafts, split-thickness skin grafts, and composite skin grafts [15]. Regarding the obstacles to skin donation in Saudi Arabia, the low number of available donors was reported as a major barrier by 2,364 (27.8%) participants. This was consistent with the findings reported in a study carried out in Turkey which reported that the lack of donor organs is the main obstacle to transplantation [22]. In contrast, a local study conducted in Riyadh reported that religion was the primary barrier to donation among the general public [19]. Regarding the esthetic outcome of skin grafting, more than half of the participants, 4,535 (53.3%), reported that the graft is similar but can be distinguished from the surrounding skin, and 1,065 (12.5%) reported that the graft is unrecognizable since it is identical to the adjacent skin. A comparative study showed similar results regarding the esthetic outcome of skin grafts [23]. Three thousand eight hundred and forty-eight (45.2%) participants reported that the distinction between the skin graft and the skin surrounding it diminishes and gradually improves with time, and this is consistent with the findings from the same study [23].

Regarding the correlation between the age of participants and the knowledge about skin donation, there was an inverse relationship between the age group and their knowledge; as the age group was older (>30 years), they reported lower scores. This might be explained by the fact that social media, which is used frequently by the young population, has played a major role in raising organ donation awareness, thus increasing the knowledge level of the young population. This is inconsistent with a previous study in which higher knowledge level was found in the older populations [19]. Our study observations revealed that female participants were more knowledgeable in comparison to male participants. This finding is inconsistent with another study that revealed that males reported higher knowledge levels than females in the local study [19]. This variation may be attributed to the fact that most of our participants were females. However, another local study conducted among medical students in Al-Majmaah supported our finding by reporting that females had a higher knowledge score compared to males [24]. Regarding educational level, uneducated responders had lower knowledge of skin donation, which is consistent with a previous study where those who had higher education expressed more enthusiasm for skin donation compared to those with lower education levels [19]. A Turkish study revealed that educated individuals were more motivated toward skin donation [22]. Similarly, the willingness to part-take in organ donation was linked to education status in similar studies from Pakistan [25]. A comparable report in Nigeria found that individuals with a higher level of education were more inclined to donate organs and skin for burn treatment [26]. Cosmetic concerns accounted for 380 (11.5%) individuals who refused skin donation, which may be explained by the concept of body distortion as it has been shown in a study that body disfigurement was the main reason for skin donation refusal [18]. In our study, the religious factor was the commonly reported hindering factor. This is consistent with a study in which the most common reason for skin donation refusal was the religious factor [19]. Additionally, this finding was identified in other local studies [20,27]. This contradicts a study carried out in Sweden, which indicated physical ugliness following donating skin tissue as the main reason for opposing skin donation [28]. Our study showed that severe burns were the most often recognized conditions that necessitate skin grafting. The finding is supported by a study in which 63.32% of the participants were aware that skin graft was used to rehabilitate burnt victims [19]. From our study, it is clear also that more than half of the respondents were willing to donate skin to close individuals, with a majority (72%) opting to replace a lost part of the skin in a visible place with another from hidden parts though from the same body. This perception did not largely agree with a study in Saudi Arabia among medical students who were likely to donate organs and educate the public about skin donation and skin banking benefits to aid burns and surgical, traumatic victims without minding the closeness of the recipient or the parts of the body where the extraction of the skin will be taken from [29].

An area of strength in this study is that the questionnaire used achieved a high response rate. Another strength is that the sample was collected from different regions of Saudi Arabia, which allows the generalization of the findings. However, the study was limited by being a questionnaire, which inherently has the risk of recall, interviewer, and response bias. Also, since most of the participants were in the younger age group, they were educated, which is majorly accredited to the nature of the online survey.

The majority of the people in Saudi Arabia seem less informed about allograft skin donation. More studies should focus on the understanding and knowledge regarding allograft skin donation in the Arab countries and explore the causation behind the withholding of skin donation, so it would be possible to address and implement the right solutions and improve the attitude of the public toward skin donation. Also, there is a need to have vigorous awareness on allograft skin donations and their importance through healthcare personnel using campaigns and direct education, and through social media which should show a major impact on the younger populations, as the case of India where information exchange has been effective in influencing people's perceptions concerning the skin donations. Hence, adequate knowledge or education will greatly change the perceptions of individuals regarding skin donations. From the study, religious factors have been pointed out as one of the main reasons why people would object to allograft skin donations; thus, to change this narrative, the religious institutions or leaders should support and encourage donations, and this has worked in countries like the Serbian Republic where religious institutions and leaders have been on the forefront to change people's perception. 

## Conclusions

Our study found that most participants support skin donation in Saudi Arabia. Religious beliefs are key determinants in influencing the perception and attitudes of respondents toward allograft skin donation. However, most respondents affirmed that their human nature was a key determinant in skin donation. Furthermore, there is a lack of knowledge of many aspects of skin donation and its role among participants. However, the study revealed that females and young participants were more knowledgeable. For the latter, it may be attributed to their exposure to social media, which had a significant role in raising organ donation awareness. Future studies should address the public perception regarding the skin graft outcome and the successfulness of skin grafts.
